# Antioxidant and Antiplatelet Activities in Extracts from Green and Fully Ripe Tomato Fruits (*Solanum lycopersicum*) and Pomace from Industrial Tomato Processing

**DOI:** 10.1155/2013/867578

**Published:** 2013-02-17

**Authors:** Eduardo Fuentes, Reinhold Carle, Luis Astudillo, Luis Guzmán, Margarita Gutiérrez, Gilda Carrasco, Iván Palomo

**Affiliations:** ^1^Department of Clinical Biochemistry and Immunohematology, Faculty of Health Sciences, Programa de Investigación de Excelencia Interdisciplinaria en Envejecimiento Saludable (PIEI-ES), Universidad de Talca, 3460000 Talca, Chile; ^2^Centro de Estudios en Alimentos Procesados (CEAP), CON ICYT-Regional, Gore Maule, R09I2001 Talca, Chile; ^3^Laboratorio de Síntesis, Instituto de Química de los Recursos Naturales, Universidad de Talca, 3460000 Talca, Chile; ^4^Institute of Food Science and Biotechnology, Chair Plant Foodstuff Technology, Hohenheim University, 70001-70619 Stuttgart, Germany; ^5^Departamento de Horticultura, Facultad de Ciencias Agrarias, Universidad de Talca, 3460000 Talca, Chile

## Abstract

The consumption of fruits and vegetables is accepted to be one of the strategies to reduce risk factors for these diseases. The aim of this study was to examine potential relationships between the antioxidant and the antiplatelet activities in green mature and fully ripe (red) tomatoes and of lycopene-rich byproducts of tomato paste processing such as pomace. The total phenol content of tomato components was the highest in peels, pulp, and in the mucilaginous myxotesta covering the tomato seeds with values 36.9 ± 0.8, 33.3 ± 00.5, and 17.6 ± 0.9 mg GAE/100 g, respectively (*P* < 0.05). Tomato peels had the highest antioxidant activity, both, as measured by the FRAP (46.9 ± 0.9 **μ**mol Fe^+2^/g, *P* < 0.05) and the DPPH assays (97.4 ± 0.2%, 1000 **μ**g/mL, *P* < 0.05). Pomace extracts showed the highest antiplatelet activity induced by ADP, collagen, TRAP-6, and arachidonic acid. While the maturation stage of the tomato fruit affected the antioxidant effect, antiplatelet activity was independent of fruit ripeness. Finally, based on the present results, tomato and its byproducts may be considered as a valuable source of antioxidant and antiplatelet activities.

## 1. Introduction

Among the nontransmissible chronic diseases (NCDS) cardiovascular diseases (CVDs) and cancer are associated with high mortality [1, 2], which is mainly due to a relative increase in the unhealthy lifestyle and the aging population [3, 4]. 

Epidemiological studies have shown that diets rich in fruits and vegetables may prevent from CVDs [5]. This protective effect might be related to their bioactive compounds [6], which has been described for fresh and processed tomatoes (*Solanum lycopersicum*) providing a cardioprotective effect through antioxidant [7] and antiplatelet activities [8] and reduction of blood lipid levels [9]. The consumption of tomato products was shown to reduce the oxidative stress induced by postprandial lipidemia and associated inflammatory response [10]. Patients suffering from atherosclerosis showed a significantly lower level of serum lycopene [11].

Pomace is a byproduct of industrial processing of tomatoes into paste and canned products. It mainly consists, of seeds and the peel [12]. However, apart from lycopene, pomace still contains other valuable compounds exerting complementary biological activities. Therefore, the evaluation of its qualitative and quantitative compositions regarding its utilization as a possible functional ingredient is of particular interest.

Consequently, the present study aimed at the comprehensive characterization of the antioxidant and antiplatelet activities of different tissues, of fully and green ripe tomatoes including the pomace resulting from industrial tomato processing.

## 2. Materials and Methods

### 2.1. Plant Material

#### 2.1.1. Tomatoes

Cluster tomatoes, green ripe and fully mature (red) tomatoes were obtained from the Regional Supply Centre of Talca, Chile, and subsequently washed and manually separated into peels, pulp, and seeds, the latter being covered by mucilage formed by its myxotesta.

#### 2.1.2. Pomace

Pomace composed of seeds and peels arising from industrial tomato paste processing was obtained from “Tresmontes Luchetti” (Production plant Talca, Chile).

### 2.2. Chemicals and Reagents

Sulfuric acid (p.a.), sodium hydroxide (p.a.), sodium carbonate (p.a.), sodium acetate (p.a.), 2,4,6-tripyridyl-s-triazine (p.a.), iron chloride (FeCl_3_), dimethyl sulfoxide (DMSO), acetylsalicylic acid, petroleum ether, methanol, ethanol, and acetone were obtained from Arquimed (Santiago, Chile), whereas lycopene, gallic acid, quercetin, catechin, Trolox (6-hydroxy-2,5,7,8-tetramethylchroman-2-carboxylic acid), butylated hydroxytoluene (BHT), 1,1-diphenyl-2-pycrylhydrazyl (DPPH), and Folin-Ciocalteu reagent were purchased from Sigma-Aldrich (St. Louis, MO, U.S.A). The agonists adenosine 5′-diphosphate bis (ADP), Thrombin receptor-activating peptide 6 (TRAP-6), and arachidonic acid were from Sigma-Aldrich (St. Louis, MO, U.S.A), while the collagen was obtained from Hormon-Chemie (Munich, Germany). Distilled water was used throughout.

### 2.3. Preparation of Extracts

#### 2.3.1. Methanol Extracts of Peel

Both, green and red peels of tomatoes were separately comminuted in a blender (Somela BL1500), and the mashed product was subsequently, mixed with methanol in a ratio of 1 g of peels to 1 mL of methanol. The mixture was sonicated (Elma Transsonic 700/H, Singen, Germany) for 5 min and then filtered through gauze twice. The filtrate was evaporated under *vacuo* (Laborota 4001, Heidolph, Germany, or RE 111-B461, Buchi Labortechnik, The Netherlands) and once concentrated, lyophilized (Freezone 6, Kansas City, Labconco, MO, USA), and stored at −80°C (Ultra Low, Sanyo Electric, Osaka, Japan) until use. 

#### 2.3.2. Aqueous Pulp Extracts

Small pieces of green and red tomatoe pulp, respectively, were comminuted in a blender, and the resulting mash was filtered twice through gauze. The liquid obtained was lyophilized and stored at −80°C until use.

#### 2.3.3. Aqueous Seed Extract

The mucilaginous myxotesta covering the tomato seeds of green and red ripe tomatoes was mixed in a ratio of 1 g of seeds to 1 mL of distilled water. The suspension was sonicated for 5 min and then filtered twice through gauze. The resulting aqueous seed extract was subsequently lyophilized and stored at −80°C until use.

#### 2.3.4. Aqueous Pomace Extract

Wet pomace was dried in an oven at 60°C for two days and subsequently comminuted and dissolved in a proportion of 1 g of tomato pomace to 1 mL of distilled water. The suspension was sonicated for 5 min and then filtered twice through gauze. The obtained aqueous tomato pomace extract was then lyophilized and stored at −80°C until use.

#### 2.3.5. Extracts of Seeds Isolated from Pomace

Seeds were recovered from pomace by sieving to obtain aqueous and petroleum ether extracts. In the first case, a portion of seeds was extracted and dissolved at a ratio of 1 g of seeds to 1 mL of distilled water, and then the mixture was sonicated for 5 min and filtered twice through gauze. The filtrate was lyophilized and stored at −80°C until use. For the petroleum ether extract, tomato pomace seeds were macerated after the addition of the solvent, and the suspension was sonicated for 5 min and then filtered twice through gauze. The filtrate was concentrated *in vacuo*, subsequently lyophilized and stored at −80°C until use.

### 2.4. Approximate Chemical Composition

To characterize the chemical composition of the pulp of green and red tomatoes, tomato pomace, and seed mucilage, the water content (%) was determined by drying in a convection oven at 60°C, the protein content was measured using the Kjeldahl method, and fat content was determined using the Soxhlet method. Ash content was obtained by drying the samples in a muffle furnace at 550°C for two hours. Crude fiber was determined by the acid sequence method using 1.25% H_2_SO_4_ and 1.25% NaOH for acid and alkaline hydrolysis, respectively. Carbohydrate content was calculated as the difference between the total and the contents of all other ingredients [13]. Each measurement was performed in triplicate.

### 2.5. Quantitation of Lycopene

Extraction and quantitative determinations of lycopene were conducted according to Fish et al. (2002) [14] using a mixture of hexane : ethanol : acetone (v/v/v 2 : 1 : 1) containing 0.05% of BHT. To avoid lycopene degradation by photooxidation and isomerization, extraction was performed under dimmed light in glassware wrapped by aluminum foil.

Quantification of lycopene for pulp of green and red tomato absorbance of the hexane extract was monitored at 503 nm using a Unicam Helios Gamma spectrometer (Thermo Spectronic, Helios Gamma, Cambridge, UK). Calibration curves of lycopene standard (*R* ^2^ = 0.99) were utilized for lycopene content determination, and results were expressed as mg/kg fresh weight. Each measurement was performed in triplicate.

### 2.6. Determination of Total Phenolic Content

Determination of total phenolic contents was performed using Folin-Ciocalteu reagent as adapted from Velioglu et al. (1998) [15], with slight modifications. In brief, 20 *µ*L of extract was mixed with 100 *µ*L of Folin-Ciocalteu reagent previously diluted with 1.58 mL of distilled water and allowed to stand at room temperature for 8 min; 300 *µ*L of sodium carbonate (20%) solution was added to the mixture. After 120 min at room temperature, absorbance was measured at 725 nm within the range of linearity (0.05–0.8 mM). Results were expressed as mg gallic acid equivalents in 100 g of the dried extract (mg GAE/100 g). Each measurement was performed in triplicate.

### 2.7. Determination of Antioxidant Activity

#### 2.7.1. DPPH Free Radical Scavenging Assay

The scavenging activity of the extracts was estimated using DPPH as the free radical model according to the method adapted from Molyneux [16]. An aliquot of 750 *μ*L of samples (100, 500, and 1000 *μ*g/mL) and control (80% methanol), respectively, was mixed with 1.5 *μ*L of DPPH. The mixture was shaken vigorously and left to stand at room temperature for 30 min in the dark. The mixture was measured spectrophotometrically at 515 nm. The free radical scavenging activity was calculated as percentage of DPPH discoloration using the following equation (1):

(1) $$\begin{array}{c} \%\;\text{scavenging}\;\text{DPPH}\;\text{free}\;\text{radical}=100\times \left(1-\frac{\text{AE}}{\text{AD}}\right), \end{array}$$

where AE is the absorbance of the solution after adding the extract at a particular level and AD is the absorbance of the blank DPPH solution. Quercetin and catechin were used as reference compounds. Each measurement was performed in triplicate. 

#### 2.7.2. FRAP (Ferric Reducing Antioxidant Power) Assay

Determinations were conducted according to Benzie and Strain [17] with modifications. The FRAP reagent was obtained by blending 300 mM acetate buffer (pH 3.6), 10 mM 2,4,6-tripyridyl-s-triazine solution, and 20 mM FeCl_3_·6H_2_O at a 10 : 1 : 1 (v/v/v) ratio prior to use and was heated to 37°C in a water bath. A total of 1.5 mL FRAP reagent was added to a test tube, and a blank reading was taken at 593 nm. A total of 50 *µ*L of selected extracts and 150 *µ*L of distilled water were added into the cuvette. After addition of the sample to the FRAP reagent, a second reading at 593 nm was performed after 90 min of incubation at 37°C. The changes in absorbance after 90 min from the initial blank reading were related to a standard curve. Standards of known Fe^2+^ concentrations were run using several concentrations ranging from 0.1 to 1 mM. A standard curve was then established by plotting the FRAP values of each standard versus their concentrations. The final result was expressed as the concentration of antioxidant exerting a ferric reducing capacity in 1 gram of sample (*µ*mol Fe^2+^/g). Trolox was used as a reference compound. Each measurement was performed in triplicate.

### 2.8. Antiplatelet Aggregation Assay

Venous blood samples were taken from two volunteers, (healthy university students), who previously signed informed consent, in 3.2% citrate tubes (9 : 1 v/v) by phlebotomy with vacuum tube system (Becton Dickinson Vacutainer Systems, Franklin Lakes, NJ, USA). The protocol was authorized by the ethic committee of the Universidad de Talca in accordance with the Declaration of Helsinki (approved by the 18th World Medical Assembly in Helsinki, Finland, in 1964). The samples were gently homogenized by 5-fold inversion and allowed to stand for 5 minutes. Then, they were centrifuged (DCS-16 Centrifugal Presvac RV) at 240 g for 10 minutes, and 1 mL of platelet-rich plasma (PRP) was taken from each tube for platelet count (in triplicate) in an hematologic counter (Bayer Advia 60 Hematology System, Tarrytown, NY, USA). The original tubes were centrifuged at 650 g for 10 minutes to obtain the platelet-depleted plasma (PDP). Finally, the PRP was adjusted to 2 × 10^5^ platelets/*µ*L with PDP. Platelet aggregation was monitored by light transmission turbidimetric method according to Born and Cross [18], using a lumi-aggregometer (Chrono-Log, Havertown, PA, USA). Briefly, 480 *μ*L of PRP in the reaction vessel was preincubated with 20 *μ*L of extract (all extracts at 1 mg/mL final reaction volume, 0.2% DMSO v/v), negative control (0.2% DMSO in final reaction volume), or positive control (acetylsalicylic acid 50 *μ*M in DMSO 0.2%). After 5 min of incubation, 20 *μ*L of agonist was added to initiate platelet aggregation, which was measured for 6 min. ADP, collagen TRAP-6, and arachidonic acid were used as agonists. All measurements were performed in triplicate. The results of platelet aggregation (maximum aggregation (%), slope, area under the curve, and lag-time (s)) were determined by the software AGGRO/LINK (Chrono-Log, Havertown, PA, USA) and the relative inhibition of the maximum platelet aggregation: 100 – ((% AgX ∗ 100)/% AgC) (% AgX: relative aggregation of the component under study, % AgC: relative control aggregation).

### 2.9. Statistical Analysis

Mean ± standard errors of mean (S.E.M) were determined using SPSS version 17.0. The data were statistically analyzed by Student's *t*-test and one-way analysis of variance using Duncan's post-hoc test. A Pearson correlation test was used to evaluate the correlation between the antioxidant activity and the phenolic compounds. The statistical significance level was set up at *P* < 0.05.

## 3. Results

### 3.1. Approximate Chemical Composition

Table 1 shows the approximate composition of red and green tomatoes, tomato pomace, and seeds. Tomato pomace is a rich source of crude fiber. As expected, seeds recovered from tomato pomace had the highest content of fat [19]. 

### 3.2. Lycopene Content

The levels of lycopene in the pulps strongly depended on the ripening degree of the green and red tomatoes. Thus, red-colored pulp of fully mature tomatoes had a more than 40 times higher lycopene content (77 ± 1.5 mg/kg fresh weight) than that in the green pulp of immature tomatoes (1.8 ± 1.2 mg/kg fresh weight) (*P* < 0.05).

### 3.3. Total Phenolic Content

**Figure 1:**
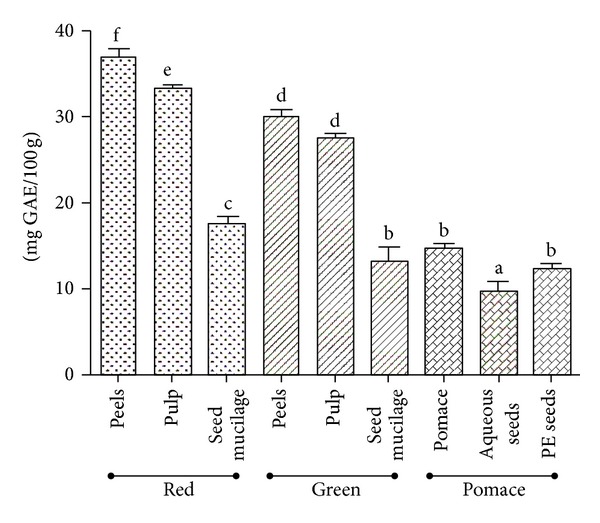
Total phenolic contents from green and fully ripe tomato fruits and pomace from industrial tomato processing. Values are presented as mean ± S.E.M (*n* = 3) which with different letters are significantly different at *P* < 0.05. Total phenolic was expressed as mg GAE acid equivalent in 100 g of dried extract. PE seeds: petroleum ether extract of seeds.

The phenolic contents deceased in the following order: peels > pulp > seeds with total contents of 36.9 ± 0.8, 33.3 ± 0.5, and 17.6 ± 0.9 mg GAE/100 g, respectively. Differences among the samples tested were significant (*P* < 0.05) (Figure 1). Peels and pulp of green tomatoes contained higher amounts of total phenolics (29.9 ± 0.8 and 27.6 ± 0.5 mg GAE/100 g, resp.) than that in the myxotesta of the seed coats (13.3 ± 1.2 mg GAE/100 g) (*P* < 0.05). Considering the different maturity degrees of the tomato fruit, the differences in polyphenol contents were significant with red tomato peels containing 36.9 ± 0.8 mg GAE/100 g) versus green tomato peels (29.9 ± 0.8 mg GAE/100 g), *P* < 0.05; and red tomato pulp (33.3 ± 0.5 mg GAE/100 g) versus green tomato pulp (27.6 ± 0.5 mg GAE/100 g), *P* < 0.05; mucilage of red tomato seeds (17.6 ± 0.9 mg GAE/100 g) versus mucilage of green tomato seeds (13.3 ± 1.2 mg GAE/100 g), *P* < 0.05 (Figure 1).

In contrast, levels of phenols in the pomace were lower than those of the green and red tomatoes. Aqueous extracts of pomace yielded higher polyphenol contents (14.6 ± 0.5 mg GAE/100 g) than petroleum ether extracts of the seeds (12.3 ± 0.5 mg GAE/100 g), while the latter were richer in polyphenols than their aqueous counterpart (10 ± 0.8 mg GAE/100 g) (Figure 1).

### 3.4. Antioxidant Activity 

#### 3.4.1. DPPH

 Generally, overall antioxidant activities were greater in all concentrations for peels and pulp followed by the seed mucilage of fully mature tomatoes. In contrast, the green tomato pulp represented the highest antioxidant activity.

**Figure 2:**
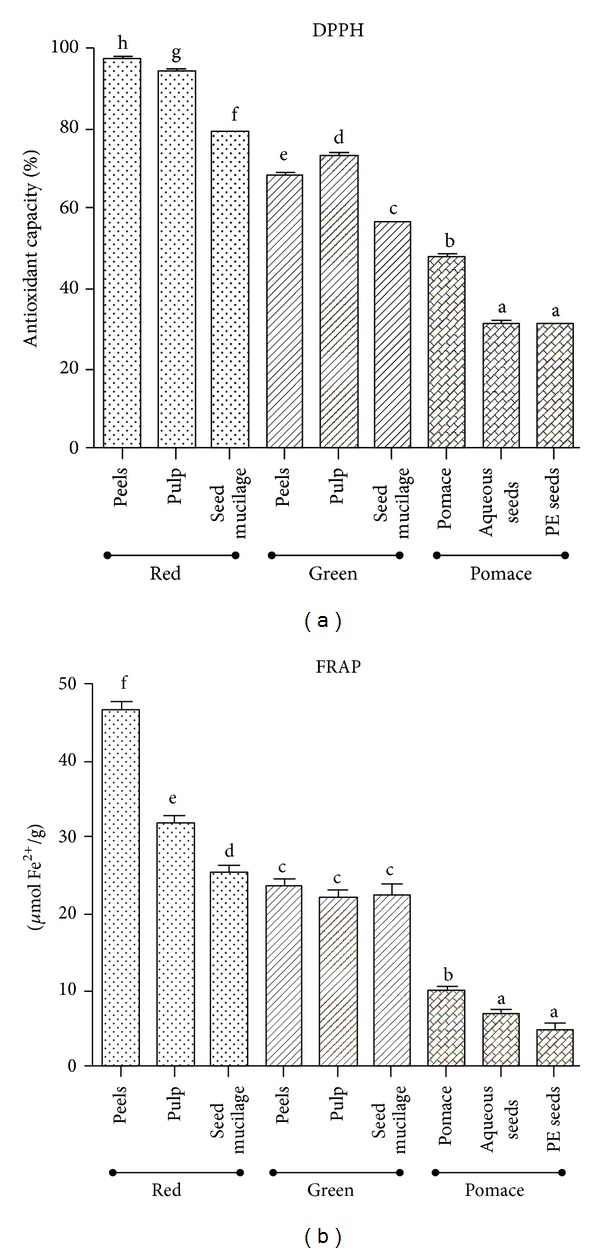
Antioxidant activity in extracts from green and fully ripe tomato fruits and pomace from industrial tomato processing. Values are presented as mean ± S.E.M  (*n* = 3) which with different letters are significantly different at *P* < 0.05. DPPH free radical scavenging activity was calculated as percentage of DPPH discoloration in 1000 *μ*g/mL of dried extract, catechin (1.8 ± 1.2 *µ*g/mL), and quercetin (1.2 ± 1.2 *µ*g/mL) showed a discoloration of 50%. Trolox (150 *µ*g/mL) presents 244 ± 1.2 *µ*mol Fe^+2^/g. PE seeds: petroleum ether extract of seeds.

When comparing different ripeness stages, the differences in their antioxidant potential were significant. At a concentration of 1000 *μ*g/mL, the value for red tomato peels amounted to 97.4 ± 0.2%, while green tomato peels showed a far lower antioxidant activity (68.5 ± 0.4%, *P* < 0.05). Red tomato pulp (94.5 ± 0.3%) was superior to green tomato pulp (73.5 ± 0.2%), *P* < 0.05 and fully mature myxotesta (79.2 ± 0.2%) showed higher values than the mucilaginous tissue of green mature tomato seeds (56.8 ± 0.2%), *P* < 0.05. Similar results were presented at 100 and 500 *μ*g/mL. Interestingly, the aqueous pomace extract tested in all study concentrations displayed a greater antioxidant activity than the aqueous and petroleum ether extracts of the seed mucilage (Figure 2).

At the highest test concentration (1000 *μ*g/mL), the results for the red tomato were in the following order: peel (97.4 ± 0.2%) > pulp (94.5 ± 0.3%) > seed mucilage (79.2 ± 0.2%) with *P* < 0.05 among the samples tested. For the green ripe tomatoes, the values were as following: pulp (73.5 ± 0.2%) > peels (68.5 ± 0.4%) > seed mucilage (56.8 ± 0.2%) with *P* < 0.05 among the samples tested. Finally, aqueous pomace extracts had a greater antioxidant activity (47.8 ± 0.3%) than the aqueous extracts (31.3 ± 0.2%) and petroleum ether extracts (30.6 ± 0.2%) of the seed mucilage (*P* < 0.05). Similar results were obtained at 100 and 500 *μ*g/mL.

#### 3.4.2. FRAP

The reducing ability was higher in extracts of red tomato than in those of green tomatoes, the latter being superior to pomace extracts. In red tomatoes, peels had higher FRAP values (46.9 ± 0.9 *µ*mol  Fe^2+^/g) than those in pulp (31.8 ± 0.9 *µ*mol Fe^2+^/g) and seeds mucilage (25.5 ± 0.8 *µ*mol Fe^2+^/g) with significant differences (*P* < 0.05) among the samples tested. In contrast, in green tomatoes, the values obtained for peels (23.6 ± 0.8 *µ*mol Fe^2+^/g) pulp (22.1 ± 0.9 *µ*mol Fe^2+^/g), and seed mucilage (22.4 ± 1.2 *µ*mol Fe^2+^/g) were similar but lower than for the corresponding tissues of the fully mature fruit. Finally, the aqueous pomace extracts showed a higher activity (9.8 ± 0.5 *µ*mol Fe^2+^/g) than the aqueous (6.9 ± 0.5 *µ*mol Fe^2+^/g) and the petroleum ether extracts (4.7 ± 0.8 *µ*mol Fe^2+^/g) of the seed mucilage (*P* < 0.05) (Figure 2).

### 3.5. Antiplatelet Activity

The results of platelet aggregation induced by the agonists ADP, collagen, TRAP-6, and arachidonic acid, respectively, with added extracts from green and fully mature tomatoes are presented in Table 2. Both extracts from green ripe and fully mature tomatoes inhibited platelet aggregation induced by ADP and collagen, respectively, but to a different extent. For the extract obtained from different tissues of red-ripe tomatoes, inhibition of platelet aggregation induced by ADP compared to control (*P* < 0.05) was in the following order: myxotesta of the seeds (65 ± 2%) > pulp (41 ± 4%) > peels (40 ± 3%). For the extracts from green ripe tomatoes, relative inhibition of platelet aggregation induced by ADP was even greater amounting to 51 ± 5% for the extract from seed myxotesta than for the pulp extract (44 ± 8%, *P* < 0.05) and peels (1 ± 1%, *P* > 0.05). Despite their different ripeness stage, the relative inhibition of platelet aggregation did not differ significantly (*P* > 0.05) for the pulp extracts from green and red tomatoes amounting to 44 ± 2 and 41 ± 3%, respectively.

Inhibition of platelet aggregation induced by collagen compared to negative control was in the following order (*P* < 0.05): extract from seed myxotesta of red ripe tomatoes (43 ± 4%) > peels (21 ± 3%) > pulp (19 ± 2%), while the extract from green tomato pulp only showed 18 ± 1% inhibition of platelet aggregation (*P* < 0.05).

Extracts from the pulp and seed myxotesta of red ripe tomatoes displayed a net lag time of 118 ± 1 and 205 ± 1 s, respectively (*P* < 0.05), in the platelet aggregation assay induced by arachidonic acid, reaching the maximum aggregation rate of >80% at 360 s.

Each of the pomace extracts exerted a potent inhibition of platelet aggregation induced by ADP, collagen, TRAP-6 and arachidonic acid, respectively (Table 3). Considering the different agonists tested in this study, the inhibition of platelet aggregation by the aqueous pomace extract was in the following order: collagen (36 ± 2%) > ADP (35 ± 3%) > TRAP-6 (22 ± 4%) > arachidonic acid (19 ± 2%) as compared to control (*P* < 0.05). When testing the aqueous extract of seed mucilage, inhibition of platelet aggregation was in the following order: collagen (80 ± 2%) > ADP (53 ± 4%) > TRAP-6 (35 ± 3%) > arachidonic acid (30 ± 4%) relative to the negative control (*P* < 0.05). Finally, the petroleum ether extract of seed mucilage inhibited platelet aggregation in the following order: collagen (80 ± 3%) > arachidonic acid (76 ± 2%) > TRAP-6 (74 ± 4%) > ADP (69 ± 3%) compared to negative control (*P* < 0.05).

## 4. Discussion

Epidemiological studies have provided evidence of a protective role of healthy diets in the prevention of CVDs and cancer [20]. Among the protective activities reported for tomato, its antioxidant activity [7] and lowering of platelet activity [21] have been associated with a decrease in the prevalence of CVDs [20].

Although pomace is a byproduct of industrial tomato processing, it presents high amount of crude fiber and protein [19]. Since it contains 13% of lysine, it is superior to soy protein, which may substantially improve the protein quality of foods low in lysine, such as cereal products [22]. Therefore, the intake of functional compounds from pomace may be associated with the reduction of inflammatory markers, blood pressure, glycemia and total cholesterol, thus decreasing cardiovascular risk [23].

The total phenolic content was higher in the red than in the green ripe tomatoes, which has been described by Ilahy et al. [24] for ordinary tomato cultivars. In tomato fruits, flavonoids (e.g., quercetin, kaempferol and naringenin) represent the major part of the total phenol content [25]. Genetic control is the main factor responsible for the accumulation of polyphenols, followed by the stages of maturation at the time of harvest, environmental factors such as light and temperature, and finally variability due to different analytical methodology [26].

The antioxidant activities as determined by the DPPH and FRAP assays were higher in red tomato followed by green tomato and tomato pomace. Although the mechanisms of action of DPPH and FRAP are different, that is, scavenging of DPPH cationic radicals in the DPPH assay and reduction of ferric ion in the FRAP assay, respectively, the results of these two assays were significantly correlated for red tomato (*r* = 0.67, *P* < 0.05) and tomato pomace (*r* = 0.78, *P* < 0.05), but not for green tomato (*r* = 0.01, *P* > 0.05). Consequently, DPPH antioxidant activity is positively correlated with polyphenol contents. This may explain higher antioxidant activity of the peels compared to the red tomato pulp, while lycopene is directly related to color measurements (red tomato > green tomato) [27]. Despite this, tomato skins are discarded by the processing industry aiming at a paste having high pulp content. Consequently, a large proportion of carotenoids is lost as waste in tomato processing [28].

The levels of total phenols showed a strong correlation with antioxidant activity as determined by the DPPH (*r* = 0.99, *P* < 0.05) and FRAP assays (*r* = 0.69, *P* < 0.05) in the red tomato. In contrast, for the extracts prepared from green fruits and pomace, only the DPPH-values were positively correlated with the total phenolics levels. FRAP noncorrelation with the antioxidant content may be due to the high content of dehydroascorbic acid in the green tomato, which might affect the sensitivity of the assay [24].

It was observed that red tomatoes exert *in vitro* [29] and *in vivo* [30] antiplatelet activity through the inhibition of platelet aggregation induced by ADP and collagen, as confirmed by our research group [21]. Recently, aqueous and methanolic extracts of red tomato were found to be thermally stable in the temperature range from 20 to 100°C and both acid and alkali did not affect the inhibition of platelet aggregation induced by ADP. The presence of lycopene in this bioactive extracts was excluded [8, 31]. Although green tomato pulp was devoid of lycopene in contrast to the pulp of red tomato, antiplatelet activity induced by ADP was similar for both ripeness stages of the fruit. Therefore, the present study confirms that lycopene cannot be made responsible for the antiplatelet activity of tomato.

Extracts prepared from the mucilaginous myxotesta of the tomato seeds exerted the greatest antiplatelet activity induced by ADP independent of the ripening stages (green and red ripe tomato). Among the tissues of the red and green tomato, the mucilage (myxotesta) covering the mature seeds exerted the maximum antiplatelet activity, which is in agreement with previous findings of Dutta-Roy et al. [29].

In food industry, pomace is obtained as a byproduct from processing tomatoes into fluid and pasty products such as tomato juice, sauce, and paste constituting a major environmental problem. It represents approximately 2% of the total weight of tomatoes processed in the agro-industry containing about 44% seeds and 56% of peels [12]. Pomace has high moisture content (> 80%) and may be used as a feed supplement for ruminants [32]. The lower antioxidant activity of the pomace may be due to the lower total phenolic content compared to red and green tomatoes [33].

Despite tomato processing under heat exposure, the active principles exerting antiplatelet activity are obviously well retained, since all the extracts were active independent of the agonist used. This may be due to the presence of lipids and *α*-tocopherol in the pomace [34]. The fat content of the seeds was found to be in the range from 15 to 30%, and 80% of the fatty acids were reported to be unsaturated, mainly comprising linoleic, oleic, and palmitic acids [35], which were shown to inhibit human platelet phospholipase A2 activity [36], thus hindering the progression of atherogenesis [37]. Linoleic acid was shown to inhibit the formation of arterial thrombosis, the expression of tissue factor, and platelet aggregation [38]. Moreover, it has been reported that *α*-tocopherol being present in the tomato pomace inhibits platelet aggregation through a PKC-dependent mechanism, which may explain a decrease in the expression of P-selectin and interactions platelet mononuclear cells *ex vivo* [39, 40]. This property is directly related to the prevention of thrombi development occurring in stroke.

The antioxidant activity was found to be dependent on the ripening degree of the tomato fruits, in contrast to the antiplatelet activity, which was also observed for extracts prepared from tomato pomace. Based on the present results, both extracts from seed mucilage and pomace may be used as functional ingredients adding antioxidant and antiplatelet activities to processed foods which may be supportive in the primary prevention of NCD.

## Figures and Tables

**Table 1:** Approximate chemical composition.

	Moisture (%)	Protein (%)	Fat (%)	Ash (%)	Carbohydrate (%)	Crude fiber (%)
Red tomato pulp	94 ± 1.5	12 ± 0.1	3 ± 0.1	15 ± 0.1	63 ± 0.4	7 ± 0.2
Green tomato pulp	95 ± 1.5	9 ± 0.1	1 ± 0.1	12 ± 0.1	71 ± 0.4	7 ± 0.2
Tomato pomace	71 ± 1.5	16 ± 0.1	2 ± 0.1	4 ± 0.1	32 ± 0.4	46 ± 0.2
Seeds of tomato pomace	9 ± 1.5	32 ± 0.1	22 ± 0.1	5 ± 0.1	25 ± 0.4	16 ± 0.1

Values are presented as mean ± S.E.M (*n* = 3). Testing procedures used in these studies were in accordance to the AOAC.

**Table 2:** Antiplatelet activity in extracts from green and fully ripe tomato fruits.

	Maximum aggregation (%)	Slope	Area under	Lag time (s)
	ADP			

Red tomato				
Peels	51 ± 0.08*	38 ± 2*	206 ± 10*	24 ± 0.03
Pulp	53 ± 0.09*	46 ± 7*	241 ± 16*	23 ± 0.01
Seed mucilage	37 ± 0.08*	38 ± 6*	149 ± 27*	33 ± 0.01
Green tomato				
Peels	86 ± 0.02	76 ± 2	319 ± 9*	21 ± 0.01
Pulp	48 ± 0.05*	47 ± 6*	205 ± 14*	19 ± 0.01
Seed mucilage	42 ± 0.08*	43 ± 12*	173 ± 15*	22 ± 0.01
Negative control	85 ± 0.02	104 ± 14*	393 ± 21	27 ± 0.01

	Collagen			

Red tomato				
Peels	71 ± 0.02*	77 ± 2*	289 ± 11	59 ± 0.01
Pulp	73 ± 0.04*	96 ± 22	300 ± 76	54 ± 0.01
Seed mucilage	51 ± 0.14*	37 ± 12*	140 ± 36	104 ± 0.01
Green tomato				
Peels	84 ± 0.04	107 ± 6	316 ± 27	60 ± 0.01
Pulp	73 ± 0.02*	63 ± 2*	222 ± 9*	84 ± 0.01
Seed mucilage	84 ± 0.09	96 ± 8*	279 ± 52*	60 ± 0.01
Negative control	90 ± 0.04	108 ± 11	284 ± 15	66 ± 0.01

	TRAP-6			

Red tomato				
Peels	80 ± 0.03	94 ± 9	394 ± 19	7 ± 0.01
Pulp	83 ± 0.05	90 ± 2*	408 ± 5	7 ± 0.01
Seed mucilage	78 ± 0.03	93 ± 7	330 ± 10	7 ± 0.01
Green tomato				
Peels	84 ± 0.02	102 ± 1	390 ± 7	10 ± 0.01
Pulp	78 ± 0.02	88 ± 2*	325 ± 1	5 ± 0.01
Seed mucilage	72 ± 0.07	87 ± 2*	310 ± 46	8 ± 0.01
Negative control	91 ± 0.01	111 ± 5	354 ± 41	10 ± 0.01

	Arachidonic acid			

Red tomato				
Peels	74 ± 0.06	71 ± 7*	313 ± 25	31 ± 0.01
Pulp	85 ± 0.06	48 ± 8*	281 ± 14	118 ± 0.01*
Seed mucilage	80 ± 0.09	61 ± 14*	154 ± 35	205 ± 0.01*
Green tomato				
Peels	76 ± 0.02	82 ± 3	276 ± 5	40 ± 0.02
Pulp	80 ± 0.09	70 ± 9	355 ± 41	23 ± 0.01
Seed mucilage	84 ± 0.06	89 ± 4	377 ± 57	34 ± 0.01
Negative control	84 ± 0.14	111 ± 12	388 ± 82	33 ± 0.01

Values are presented as mean ± S.E.M (*n* = 3). ADP 8 *μ*M, collagen 1.5 *μ*g/mL, TRAP-6 30 *μ*M, and arachidonic acid 1 mM. Extracts at 1 mg/mL. *P < 0.05 versus negative control (saline 0.9%).

**Table 3:** Antiplatelet activity in extracts from pomace.

	Maximum aggregation (%)	Slope	Area under	Lag time (s)
	ADP			

Pomace	55 ± 0.12*	56 ± 6*	220 ± 31*	31 ± 0.01
Aqueous seeds	40 ± 0.09*	39 ± 7*	152 ± 30*	19 ± 0.01
PE seeds	26 ± 0.04*	29 ± 9*	103 ± 10*	21 ± 0.01
Negative control	85 ± 0.02	104 ± 14	393 ± 21	27 ± 0.01

	Collagen			

Pomace	58 ± 0.05*	66 ± 9*	218 ± 32	64 ± 0.01
Aqueous seeds	10 ± 0.01*	12 ± 1*	39 ± 3*	52 ± 0.01
PE seeds	18 ± 0.03*	21 ± 3*	65 ± 6*	66 ± 0.01
Negative control	90 ± 0.04	108 ± 11	284 ± 15	66 ± 0.01

	TRAP-6			

Pomace	71 ± 0.04*	86 ± 7*	337 ± 40	13 ± 0.01
Aqueous seeds	59 ± 0.06*	67 ± 3*	264 ± 29	5 ± 0.01
PE seeds	23 ± 0.03*	25 ± 3*	95 ± 3*	16 ± 0.01
Negative control	91 ± 0.01	111 ± 5	354 ± 41	10 ± 0.01

	Arachidonic acid			

Pomace	68 ± 0.05*	57 ± 11*	289 ± 30	47 ± 0.01
Aqueous seeds	59 ± 0.11*	66 ± 10*	289 ± 94*	28 ± 0.01
PE seeds	20 ± 0.05*	18 ± 3*	92 ± 18*	18 ± 0.01
Negative control	84 ± 0.14	111 ± 12	388 ± 82	33 ± 0.01

Values are presented as mean ± S.E.M (*n* = 3). ADP 8 *μ*M, collagen 1.5 *μ*g/mL, TRAP-6 30 *μ*M, and arachidonic acid 1 mM. Extracts at 1 mg/mL. *P < 0.05 versus negative control (saline 0.9%). PE seeds: petroleum ether extract of seeds.
